# Interval estimation of disease loci: development and applications of new linkage methods

**DOI:** 10.1186/1471-2156-6-S1-S21

**Published:** 2005-12-30

**Authors:** Charalampos Papachristou, Shili Lin

**Affiliations:** 1 Department of Statistics, The Ohio State University, 1958 Neil Avenue, Columbus, OH 43210, USA

## Abstract

Three variants of the confidence set inference (CSI) procedure were proposed and applied to both the simulated and the Collaborative Study on the Genetics of Alcoholism (COGA) data. For each of the two applications, we first performed a preliminary genome scan study based on the microsatellite markers using the GENEHUNTER+ software to identify regions that potentially harbor disease loci. For each such region, we estimated the sibling identity-by-descent sharing probability distribution at the putative disease locus. Based on these estimated probabilities, the CSI procedures were employed to further localize the disease loci using the single-nucleotide polymorphism markers, leading to confidence intervals/regions for their locations. For our analysis with the simulated data, we had knowledge of the simulating models at the time we performed the analysis.

## Background

A frequently used strategy in linkage analysis is to first screen the entire genome using microsatellite (MS) markers, and then to follow up on preliminary linkage regions using densely saturated (often single-nucleotide polymorphisms (SNP)) markers. Although many statistical methods are available for each step of this two-stage approach, only a limited number of them (e.g., Liang et al. [1]) are able to provide confidence estimates of disease gene locations. Furthermore, most of the methods available are subject to multiplicity adjustment, which is a non-trivial matter given the complex dependency of the statistics involved.

The confidence set inference (CSI) procedure [2] can be used to obtain confidence estimates using affected sib-pair (ASP) data, and avoids the multiple testing problem. Unlike the approach of Liang et al. [1], it is not based on the asymptotic distribution of the estimator of the location of the trait locus. Instead, it indirectly deduces a confidence region for the trait locus based upon a set of markers that are inferred to be within a pre-specified distance from the trait locus. Note that this is a non-directional procedure that makes no distinction between loci symmetrically located around a marker. In the present paper, we explore three variants of the CSI procedure to further improve its performance. The first modification is to test every location (not just the markers) in the region of interest. This practice effectively eliminates the "non-directional" problem of the original CSI method. The second variant is a multipoint extension of the first variant, in that information from all markers are utilized to calculate the IBD sharing statistic at a marker locus. The last is also a multipoint approach, but the identity-by-descent (IBD) sharing statistic is calculated at each hypothesized disease locus rather than at its nearest marker locus.

## Methods

### Confidence set inference

This is a two-point approach that tests the following hypotheses for each marker *m*:

H_0m _: θ_m _≤ θ_0 _vs. H_am_: θ_m _> θ_0_,

where *θ*_*m *_denotes the true, but unknown, recombination fraction between the disease locus and marker *m*, and *θ*_0 _is a predetermined recombination fraction. Note that the above null and alternative hypotheses are the reversals of those in traditional linkage analysis. It is actually this formulation that allows us to construct the confidence region for the location of the trait locus [2,3]. For testing the above hypotheses with ASP data, we use the mean statistic [4] because it has been shown to perform well under a wide variety of disease models [3,4]. Recently, Elston et al. [5] suggested that, when there is actually no linkage, the average IBD sharing between sib pairs in an ASP-only design maybe higher than what would be expected under the traditional null hypothesis of no linkage. Because our null hypothesis assumes tight linkage, the effect of such phenomenon on our method is unclear, and thus further investigation is needed.

Let *L *be the set of markers for which the corresponding null hypotheses are not rejected at level α. Then the probability that *L *includes at least one marker located within *θ*_0 _from the disease locus is at least (1 – *α*). From this set of markers, we can deduce the following confidence region for the disease locus:



where *τ*_*m *_is the map position of marker *m*, while *d (θ*_0_) is the genetic distance that corresponds to recombination fraction *θ*_0_. In practice, *θ*_0 _is usually chosen to correspond to half of the maximum distance between any two adjacent markers in the region to be investigated.

### Variant I (CSI-v1): testing at an arbitrary locus

For an arbitrary location *τ *in the preliminary linkage region, we test the following hypotheses:

*H*_0*m *_: *τ *= τ* *vs. H*_*am *_: *τ *≠ τ*

where τ* is the true location of the disease locus in the region, and *m *is the marker closest to *τ*. The following is our strategy for carrying out these tests. First, we consider a finite number of loci (say, at 1 cM density) in the region. For each of these loci, we test whether the IBD sharing at the nearest marker locus *m *is within the margin of error from what is expected if the disease locus is indeed at *τ *(the null hypothesis). We then iteratively refine the discretization strategy so that the locations for which the null hypotheses are not rejected constitute a (union of) "continuous" chromosomal segment(s).

### Variant II (CSI-v2): multipoint extension

This is the multipoint extension of CSI-v1. The hypotheses and the discretization/ search strategies are the same as before. However, when calculating the observed sharing statistic at the nearest marker locus *m*, information from all markers, not just marker *m *itself, are used.

### Variant III (CSI-v3): multipoint extension with sharing statistic

This is another multipoint extension with the same hypotheses as in CSI-v1 and CSI-v2, but the observed sharing statistic is calculated at the hypothesized locus itself given all the observed marker data.

### Estimation of IBD sharing probabilities at putative disease loci

In Papachristou and Lin [2], the IBD sharing probabilities are estimated through the use of population risk characteristics (disease prevalence, relative risks for offspring and sibling), which are frequently available from population epidemiological studies. Alternatively, these probabilities can be estimated directly from the current data after preliminary linkage regions are established. Let *τ *be the putative disease locus (at which the maximum score occurs) in a linkage region, and *z*_*k*_, *k *= 0, 1, 2, be the probabilities that an ASP shares *k *alleles IBD at *τ*. Then, the likelihood of *z*_*k *_is



where *n *is the number of ASPs in the study. Note that the likelihood is parameterized in terms of *z*_*1 *_and *z*_*2 *_only, since *z*_0 _is completely determined by the other two. For more details on the computation of the above likelihood the reader is referred to Kruglyak et al. [6]. The *z*_*k *_values that maximize the above likelihood (obtained using the EM algorithm) are taken as estimates of the IBD probabilities.

### Data and phenotypes

For all four simulated populations, we extracted all possible families with at least two affected children. For families with three or more affected children all possible pairs were formed and were treated as if they were independent nuclear families. This method yielded an average of 150, 170, 150, and 180 ASPs for the AI, DA, KA, and NYC populations, respectively. For the application to the Collaborative Study on the Genetic Analysis of Alcoholism (COGA) data, ASPs were also extracted from the extended pedigrees, yielding a sample of 551 pairs. The ALDX1 diagnostic criterion was used, and only those who were confirmed to be affected were used in our study.

### Selection of SNPs in the linkage regions

After linkage regions are identified using the method of Kong and Cox (KAC) [7] as implemented in GENEHUNTER+, a variant of GENEHUNTER [6], SNPs surrounding the maximum KAC scores are selected for CSI analyses. For chromosome 1 in the simulated data, we use 20 SNP markers, ten on each side of the maximum KAC score, that cover a region of about 60 cM. Because the regions on chromosomes 5 and 9 are usually maximized at the beginning of the chromosomes, we use the first 15 available SNP markers that are spread over a region of about 55 cM. On the other hand, the linkage regions on chromosome 3 are usually maximized at the end of chromosome 3, so we use the last 15 SNP markers of the chromosome that spanned a region of 45 cM. For the COGA data, for each chromosome with a maximum KAC score exceeding the threshold, we used all the Illumina SNPs within 30 cM (or less if the end of a chromosome is encountered as in chromosome 12) on each side of the maximum score for CSI analyses.

## Results

### Simulated data

First, the KAC scores were calculated using the MS markers throughout the whole genome. Only chromosomes 1, 3, 5, and 9 yielded significant results using the threshold of 3.09 (corresponding to a pointwise significant level of 0.001) for more than one-third of the replicates in any of the populations. Therefore, we decided to focus on those 4 chromosomes for obtaining confidence regions using CSI and its variants based on the 3-cM-density SNPs.

From the clinical ascertainment scheme, DA would be most informative for a locus influencing the behavioral symptoms. It turns out that, in the simulating model, locus D1 (on chromosome 1) plays an important role in the trait relating to these symptoms. Indeed, the KAC results reveal that only population DA has a majority of the replicates (in fact, all 100 of them) showing linkage at the pointwise significance level of 0.001. Among the other three populations, only AI has more than one-third of the replicates (45) showing significant results. Similarly, as expected from the ascertainment schemes, all four populations contain information about the disease gene on chromosome 3, and thus they all have more than one-third of the replicates (61, 82, 39, and 50 for AI, DA, KA, and NYC, respectively) with significant results. For the genes on chromosomes 5 and 9, on the other hand, only AI and KA contain information about their locations. Again, the results are consistent with the simulating model in that KA is more informative about these two loci than AI (77 vs. 48 for D3, and 76 vs. 36 for D4). Surprisingly, more than two-thirds of the replicates from NYC are not informative for these loci.

Table 1 presents the results from the four CSI procedures with a preset 95% coverage probability. For each disease locus, only populations that are informative for linkage for at least one-third of the replicates are investigated. As can be seen from the results, the original CSI method indeed produced wide regions. On the other hand, the two multipoint variants (CSI-v2 and CSI-v3) have considerably narrower regions, with almost all of them including the true disease locations.

### The COGA dataset

**Figure 1:**
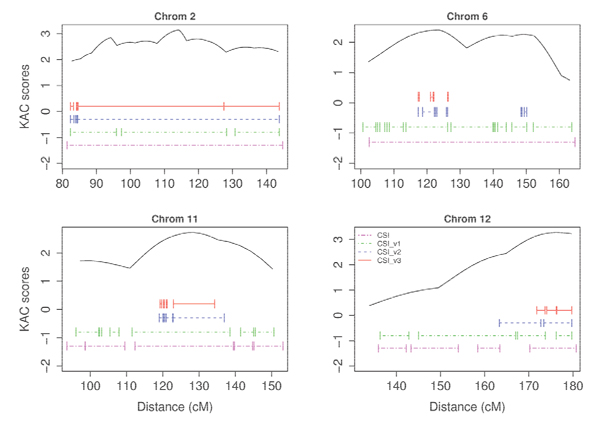
**Results from the COGA dataset. **The curve on each plot represents the KAC scores (using MS markers) in the preliminary linkage regions plotted again the SNP map. The four confidence regions (line segments) plotted below the curve are the results from the four CSI procedures using the SNPs, as identified in the legend of the figure. In particular, the confidence regions inferred from CSI-v3 for chromosomes 2, 6, 11, and 12 are, respectively, C2 = [82.4, 83.3] ∪ [84.1, 84.2] ∪ {84.4} ∪ [84.6, 127.4] ∪ [127.5, 143.8], C6 = [117.3, 117.7] ∪ [121.1, 121.8] ∪ [121.9, 122.1] ∪ [126.2, 126.4], C11 = [119.3, 119.7] ∪ [120.2, 120.4] ∪ [121.0, 121.2] ∪ [122.9, 134.3], and C12 = [171.8, 173.7] ∪ [174.1, 176.2] ∪ [176.3, 179.7]

Whole-genome screening using KAC based on the MS markers resulted in 6 chromosomal regions (one of each on chromosomes 1, 2, 6, 11, 12, and 15) with the maximum KAC scores exceeding the cutoff of 2.33 (a pointwise significant level of 0.01). As with the simulated data, our analysis scheme, after preliminary linkages are established, is to use the SNPs data to further narrow down the linkage regions. Specifically, the four CSI procedures, all with 95% coverage probability, were used to construct confidence regions for the disease gene locations. However, chromosomes 1 and 15 are not included in the CSI analysis as there are no SNPs in the preliminary linkage regions (toward the ends) on these two chromosomes. Figure 1 shows the results for the remaining four chromosomes focusing on the linkage regions. For chromosomes 6, 11, and 12, the results demonstrate the abilities of the two multipoint CSI methods for narrowing down the linkage regions. In particular, CSI-v3 narrows the regions on these three chromosomes to 1.5, 12.3, and 7.4 cM, respectively. However, for chromosome 2, the two multipoint procedures failed to narrow further from the two-point regions. By inspecting the KAC scores in the region, it appears that there are potentially multiple disease loci in the region, which might, in part, explain the CSI results.

## Discussion

The purpose of this contribution is two-fold. First, we want to demonstrate that, unlike most of other linkage methods, confidence regions with pre-specified coverage probabilities can be obtained by the CSI procedures. This is especially useful following preliminary linkage analysis. Specifically, after linkage is established, dense SNP markers can be genotyped in the linkage regions so that the CSI procedures can then be applied, perhaps as an intermediate mapping method before fine mapping association studies commence. Second, through the analyses of both the simulated and the COGA data, we show that the CSI procedure [2] can be further refined to provide narrower confidence regions for disease gene locations. We are highly encouraged by the extremely high actual coverage probabilities for the two multipoint CSI procedures, as can be seen from the simulated data. This would also give us confidence in results from real applications.

For the COGA dataset, we are able to place the disease loci on three of the chromosomes to narrow confidence regions using CSI-v3 (ranging from 1.5 cM to 12.3 cM in length), which may have potential implications in studying the genetics of alcoholism. For the simulated data, however, the confidence regions are still quite large (around 30 cM for most of them) with the two multipoint variants. We speculate that this is mainly due to the limited informativeness of the still quite sparse 3-cM-density SNP markers. We believe that, with a much denser SNP map (say one with 0.25–0.5 cM inter-marker separation), further narrowing can be achieved. Moreover, we also plan to explore other methods for estimating relative risks (or IBD probability distributions at disease loci) to examine their effects on the results from the CSI procedures.

## Abbreviations

ASP: Affected sib-pair

COGA: Collaborative Study on the Genetics of Alcoholism

CSI: Confidence set inference

EM: Expectation maximization

IBD: Identity-by-descent

KAC: Kong and Cox

MS: Microsatellite

SNP: Single-nucleotide polymorphism

## Authors' contributions

Both authors contributed equally to the conceptual development of the project. CP performed the analysis. SL drafted the manuscript. Both authors read and approved the final manuscript.

## Figures and Tables

**Table 1:** Percentages^a ^of CSI confidence regions including disease genes. The average length (L) and the standard deviation (SD) of the lengths of the confidence regions are also provided.

		Population											
		
		AI			DA			KA			NYC		
		
Gene	Method	%	L	SD	%	L	SD	%	L	SD	%	L	SD
D1	CSI	100	59.8	7.2	100	54.5	8.1	-	-	-	-	-	-
	CSI-v1	93	55.6	7.5	95	46.8	8.7	-	-	-	-	-	-
	CSI-v2	100	48.4	12.8	99	36.4	12.8	-	-	-	-	-	-
	CSI-v3	100	49.2	12.6	97	37.0	12.2	-	-	-	-	-	-

D2	CSI	97	39.5	5.8	99	38.0	5.8	100	39.4	5.3	98	37.5	6.0
	CSI-v1	92	36.1	7.2	91	34.4	6.9	95	36.5	6.4	88	34.1	7.5
	CSI-v2	100	29.2	10.8	99	26.3	8.6	100	29.9	9.6	98	28.8	10.1
	CSI-v3	100	29.8	10.8	100	26.9	8.4	100	30.7	9.4	98	29.0	9.8

D3	CSI	98	39.3	5.8	-	-	-	100	38.6	4.7	-	-	-
	CSI-v1	92	36.9	5.6	-	-	-	100	36.1	5.3	-	-	-
	CSI-v2	100	31.2	9.8	-	-	-	100	28.9	10.4	-	-	-
	CSI-v3	100	31.7	9.5	-	-	-	100	29.2	10.4	-	-	-

D4	CSI	94	39.2	5.6	-	-	-	96	38.6	5.1	-	-	-
	CSI-v1	94	36.8	5.9	-	-	-	93	36.0	5.2	-	-	-
	CSI-v2	100	31.5	9.5	-	-	-	100	29.9	8.5	-	-	-
	CSI-v3	100	31.7	9.4	-	-	-	100	30.1	8.5	-	-	-

^a ^These are percentages of replicates among those that have been inferred to be linked by KAC at the threshold of 3.09. For each disease gene, if data from a particular population did not lead to inferences of significance linkage for at least one-third of the replicates, the results for that population are not included.
